# Beyond incidence: clarifying the evidence-based predictors of failed spinal anesthesia

**DOI:** 10.1186/s13037-025-00471-x

**Published:** 2026-01-29

**Authors:** Tuhin Mistry, Abhijit Sukumaran Nair

**Affiliations:** 1 Department of Anaesthesiology and Perioperative Care, Ganga Medical Centre & Hospitals Pvt Ltd, Coimbatore, India; 2 Department of Anaesthesiology, Ibra Hospital, Ibra, Sultanate of Oman

Dear Editor,

We read with interest the systematic review and meta-analysis by Zegeye et al., which attempts to estimate the global incidence and predictors of failed spinal anesthesia (SA) [1]. The authors address a clinically relevant topic; however, several methodological issues limit the interpretability and generalisability of the findings.

A major concern is the lack of uniformity in how ‘failure’ was defined across the included studies. Outcomes ranged from inadequate sensory block to the need for supplementation or conversion to general anesthesia, yet these distinct endpoints were pooled as a single outcome. Contemporary neuraxial literature emphasises the importance of distinguishing partial, patchy, and complete failure, as these entities differ mechanistically and clinically [2–4]. Without harmonised definitions, the reported pooled incidence of 8.36% becomes difficult to interpret and likely misrepresents true clinical failure rates.

Interpretation of several predictors reported in the meta-analysis also requires caution. Variables such as body mass index, emergency surgery, limited provider experience, and the number of puncture attempts are inherently interrelated. Without multivariable adjustment, associations may reflect confounding rather than independent effects. Prospective studies have repeatedly demonstrated that anatomical difficulty and operator skill are major determinants of SA success, often mediating the influence of factors like obesity or urgency of surgery [5, 6]. Likewise, the reported increased risk associated with isobaric bupivacaine contradicts high-quality evidence from randomized trials and meta-analyses demonstrating comparable efficacy between hyperbaric and isobaric formulations when dosing, positioning, and technique are standardised [7–9]. These inconsistencies likely reflect study-level variability rather than intrinsic pharmacological differences.

A DerSimonian–Laird random-effects model was used to estimate the pooled incidence [1]. However, there is no mention of a variance-stabilizing transformation such as the Freeman–Tukey double–arcsine or the generalized linear mixed-model approach [10]. Given the extreme heterogeneity reported (I² ≈ 98.6%), pooling raw proportions with a traditional random-effects method risks producing biased prevalence estimates and inaccurate confidence intervals, particularly when true proportions vary widely across studies. Additionally, although the authors state that adjusted odds ratios were log-transformed before pooling, it remains unclear whether all included effect sizes were adjusted consistently or whether adjusted and unadjusted estimates were combined. Mixing effect sizes derived under different confounding structures limits interpretability and risks distorting causal inference. Explicit reporting of adjustment variables and separate pooling of adjusted versus unadjusted estimates would significantly strengthen the validity of the findings.

Moreover, the extremely high heterogeneity (I² > 98%) further challenges the meaningfulness of a single pooled incidence [1]. Obstetric, orthopedic, and mixed surgical cohorts differ significantly in their physiological profiles, block height requirements, and surgical duration. Prior multicenter investigations highlight that obstetric SA failures often relate to anatomical factors or inadequate block assessment, whereas orthopedic cases fail due to insufficient spread for higher dermatomal levels [11, 12]. Although the authors performed subgroup analyses, surgery-specific pooled estimates were not presented, limiting clinical applicability. Additional findings, such as the markedly elevated risk associated with previous anesthesia or with puncture at the L4–L5 level, require careful contextualisation, as neither is an established independent predictor in contemporary practice unless confounded by prior spine surgery, difficult anatomy, or institutional preference patterns.

Finally, the predominance of studies from resource-limited settings introduces further considerations. Differences in provider training, equipment availability, case complexity and workload may inflate the pooled incidence compared with high-resource environments, limiting global generalisability. Recent studies continue to show wide geographic variability in SA failure rates, underscoring the need for context-specific interpretation [9, 11].

To conclude, while the review draws attention to the multifactorial nature of SA failure, methodological heterogeneity, particularly in outcome definitions, population mixing, and unadjusted predictors analyses limit the strength of its conclusions. Future syntheses would benefit from standardised definitions of failure, stratified analyses by surgical population, and multivariable-adjusted effect estimates to provide more clinically meaningful guidance.

## Data Availability

No datasets were generated or analysed during the current study.
